# Supplementary material for: Prior Infection by Colletotrichum spinaciae Lowers the Susceptibility to Infection by Powdery Mildew in Common Vetch
Source: Plants (Basel). 2023 Dec 22;13(1):52. doi: 10.3390/plants13010052 (PMC10780821; doi:10.3390/plants13010052)
Supplement: Supplementary file 1 [file plants-13-00052-s001.zip › plants-2768908-supplementary.pdf]

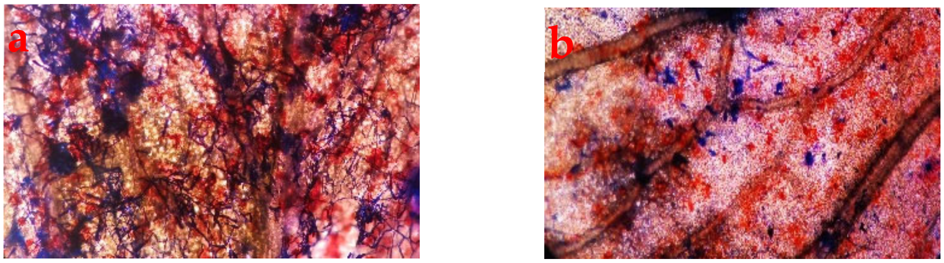

Figure S1. Morphological structures of powdery mildew observed by commassie brilliant blue staining. (**a**) mycelium structure of C^{-} plant leaf; (**b**) mycelium symptoms of C^{+} plant leaf.

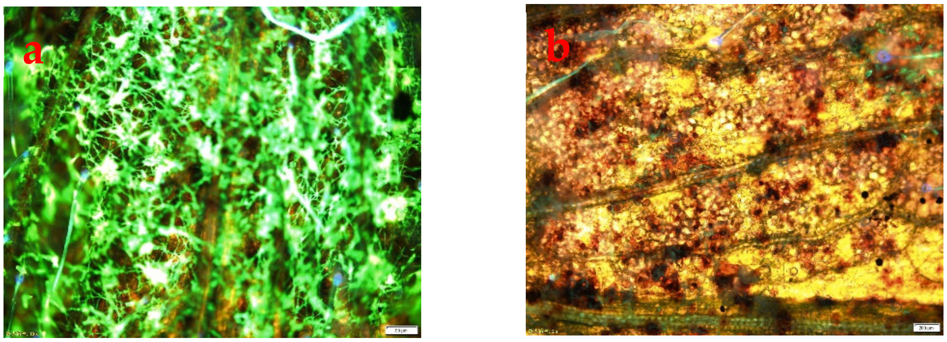

Figure S2. Morphological structures of powdery mildew observed by uranine fluorescence staining. (**a**) mycelium structure of C^{-} plant leaf; (**b**) mycelium symptoms of C^{+} plant leaf.
